# Onychomadèse des 20 ongles

**DOI:** 10.11604/pamj.2014.18.303.5022

**Published:** 2014-08-15

**Authors:** Fatima Zahra Elfatoiki, Soumia Chiheb

**Affiliations:** 1 Service de Dermatologie, CHU Ibn Rochd, Casablanca, Maroc

**Keywords:** Onychomadese, syndrome main pied bouche, Nourrisson, onychomadesis, foot mouth hand syndrome, infant

## Image en medicine

Nourrisson de 5 mois adressé en dermatologie pour atteinte unguéale des 20 ongles évoluant depuis un mois. Cette symptomatologie était apparue 3 semaines au décours d'une éruption fébrile a type d'exanthème vésiculeux à prédominance péribuccale et palmoplantaire ayant régressé sous simple traitement symptomatique. L'examen clinique retrouvait une onychomadèse de la majorité des ongles. L'examen cutané notait une légère desquamation de l'extrémité des doigts et des orteils. Par ailleurs l'enfant était apyrétique, en bon état général. Une abstention thérapeutique était proposée avec une bonne évolution de l'atteinte unguéale. Le recul est de 2 mois.

**Figure 1:**
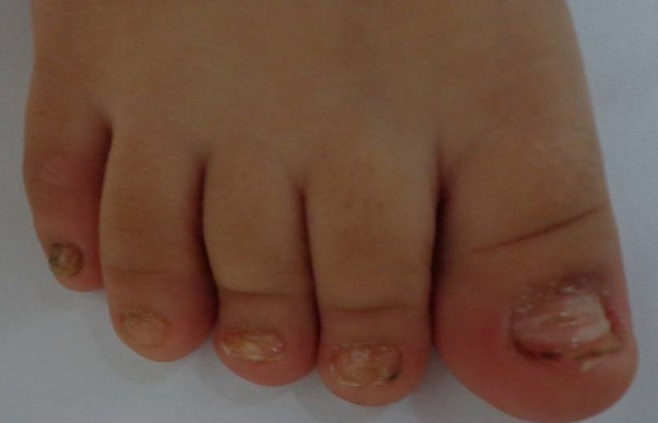
Onychomadèse dans le cadre d'un syndrome main pied bouche. Atteinte unguéale des orteils du pied

